# From Molecular Pathophysiology to Clinical Trial Design in Sjögren’s Disease: A Three-Axis Framework

**DOI:** 10.3390/ijms27135692

**Published:** 2026-06-24

**Authors:** Muhammad Soyfoo, Julie Sarrand, Christine Delporte

**Affiliations:** 1 Department of Rheumatology, Hôpital Erasme, Université Libre de Bruxelles (ULB), Route de Lennik 808, 1070 Brussels, Belgium; julie.sarrand@ulb.be; 2 Laboratory of Pathophysiological and Nutritional Biochemistry, Université Libre de Bruxelles (ULB), 1070 Brussels, Belgium; christine.delporte@ulb.be

**Keywords:** Sjögren’s disease, precision medicine, biomarkers, interferon signature, CXCL13, ianalumab, patient stratification, clinical trial design, B-cell hyperactivity, translational rheumatology

## Abstract

Sjögren’s disease (SjD) remains one of the few major systemic autoimmune diseases without an approved disease-modifying therapy, despite decades of pathogenic insight and several randomised trials. We contend that these repeated failures reflect not intrinsic therapeutic refractoriness, but trial designs insufficiently aligned with the underlying biological heterogeneity of SjD. We propose a tripartite framework in which SjD is organised around three dominant biological axes: an interferon-driven systemic axis, a B-cell/lymphoproliferative axis, and a symptom/fibro-structural axis. Each axis carries its own characteristic biomarkers, histopathology, prognostic features, candidate endpoints, and therapeutic targets, and each implies a distinct trial enrolment strategy. Recent positive trials—phase III for ianalumab in NEPTUNUS-1/2, phase 2b for iscalimab in TWINSS, phase 2 for nipocalimab in DAHLIAS, and phase 2 for dazodalibep in a phenotype-defined symptom-dominant cohort—illustrate that meaningful clinical benefit becomes detectable once stratification is aligned to biology. By integrating molecular endotypes, validated biomarkers, composite endpoints, and phenotype-matched therapies onto a single explicit architecture, SjD shifts from a recurring example of translational failure to a model for precision medicine in heterogeneous autoimmune disease. The central message is that SjD may be less intrinsically treatment-resistant than it has historically been treatment-mistargeted.

## 1. Introduction

Sjögren’s disease (SjD) is a chronic systemic autoimmune disease affecting approximately 0.3–1% of the population with a marked female predominance [1,2]. Its clinical hallmark is sicca symptomatology arising from lymphocytic infiltration of exocrine glands, but the disease extends well beyond glandular involvement: up to 40% of patients develop systemic manifestations including inflammatory arthritis, interstitial lung disease, peripheral neuropathy, or cutaneous vasculitis [3,4,5]. The cumulative burden of sicca, fatigue, and pain substantially impairs functional status and quality of life [6,7,8,9], and patients carry the highest lifetime risk of B-cell non-Hodgkin lymphoma among systemic autoimmune diseases—a 15- to 20-fold excess over the general population [3]. Throughout this review we use the contemporary term “Sjögren’s disease” (SjD) in preference to the older “Sjögren’s syndrome”, reflecting current nomenclature that recognises the condition as a systemic autoimmune disease in its own right rather than merely a secondary syndrome; we treat the two terms as synonymous and retain “Sjögren’s syndrome” only where it forms part of the established name of a cited instrument, trial, classification criterion, or historical source. This biological burden is compounded by a clinical one that any bench-to-bedside framework must ultimately address: diagnosis is frequently delayed by several years because early symptoms are non-specific and confirmatory testing is unevenly accessible, and the gap between rapidly expanding molecular insight and day-to-day clinical decision-making remains wide. The framework proposed here is therefore intended not only to organise SjD biology but to make that biology actionable at the level of diagnosis, stratification, and treatment selection in females.

Despite this burden, SjD remains without an approved disease-modifying therapy. Current EULAR recommendations explicitly acknowledge this gap [10], and management remains symptomatic [11,12,13]. The trial record is strikingly extensive. Large, well-designed randomised controlled studies of rituximab (TRACTISS, TEARS) [14,15] and abatacept (ASAP-III) [16] failed to meet their primary endpoints despite strong mechanistic rationale, and they did so in the context of more than thirty further negative trials. The conventional reading of this record—that SjD is somehow intrinsically resistant to immunomodulation—is, in our view, the wrong reading. The negative trials share three structural features: biologically heterogeneous populations enrolled by classification criteria rather than disease biology, endpoints that conflate inflammation with established structural damage, and therapeutic mechanisms applied without reference to a dominant pathway in the patient enrolled. Where any of these has been corrected, signal has emerged. Ianalumab in the replicate phase III NEPTUNUS-1 and NEPTUNUS-2 trials met the ESSDAI primary endpoint, enrolling patients with documented systemic activity [17,18,19]; iscalimab achieved a dose–response on ESSDAI in the TWINSS phase 2b study [20]; nipocalimab produced an early signal in the DAHLIAS phase 2 trial [21]; and dazodalibep showed efficacy specifically in a prospectively defined symptom-dominant subset [22]. The pattern is consistent: biology-aware design produces detectable benefit; biology-naïve design does not.

This review proposes that SjD is best understood as a composite of three dominant biological axes: an interferon (IFN)-driven systemic axis, a B-cell/lymphoproliferative axis, and a symptom/fibro-structural axis. The axes are not mutually exclusive—most patients show features of more than one—but the dominant axis differs across patients in ways that should determine biomarker selection, endpoint choice, and therapeutic targeting. We argue this framework throughout, mapping pathogenesis (Section 2), patient stratification (Section 3), translational biomarkers (Section 4), therapeutics (Section 5), and trial roadmap (Section 6) onto the same three-axis architecture. The objective is to convert what has been an unstructured catalogue of heterogeneity into a working clinical and translational ontology.

Search strategy and selection criteria. This is a narrative, non-systematic review. We searched PubMed/MEDLINE from inception to October 2025 using combinations of “Sjögren’s syndrome” or “Sjögren’s disease” with “precision medicine”, “biomarker”, “stratification”, “endotype”, “interferon signature”, “CXCL13”, “BAFF”, “ianalumab”, “iscalimab”, “nipocalimab”, “dazodalibep”, “rituximab”, “abatacept”, “ESSDAI”, “ESSPRI”, “STAR”, and “CRESS”. Reference lists of pivotal trials, EULAR/ACR position papers, and recent reviews were hand-searched. We prioritised peer-reviewed publications, randomised controlled trials, and large prospective cohorts; pivotal congress abstracts (ACR 2025, EULAR 2025) were retained when relevant peer-reviewed data were not yet available. Selection was guided by translational relevance rather than exhaustive coverage and was reached by author consensus. The search was restricted to English-language records. Preprints were not included; only peer-reviewed publications and selected congress abstracts were considered. Congress abstracts were eligible only when they reported pivotal or late-breaking trial data (ACR 2025, EULAR 2025) for which no corresponding peer-reviewed publication was yet available, and such sources are explicitly identified as abstract-level evidence in the text and in Table 1. Records were screened independently by two authors, and disagreements were resolved by discussion and consensus with the senior author. We emphasise that this review is explicitly narrative and not systematic: it was not conducted or reported in accordance with PRISMA, no formal protocol was registered, and no formal risk-of-bias or quality assessment of included studies was performed. The synthesis is therefore interpretive rather than quantitative, and the strength of the underlying evidence is graded qualitatively throughout the text and in Table 1 rather than through a structured appraisal instrument. 

## 2. Three Biological Axes of Sjögren’s Disease

**Figure 1:**
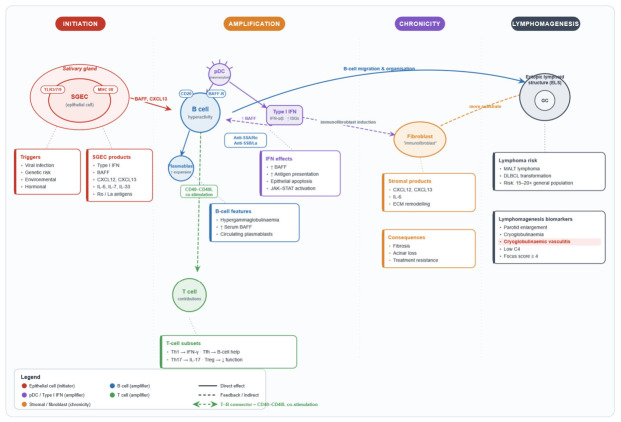
Schematic representation of the mechanistic events organised by temporal and functional roles. Initiation: salivary gland epithelial cells (SGECs) function as active participants through expression of Toll-like receptors (TLR3/7/9), MHC class I/II molecules, and production of type I interferons, BAFF, chemokines (CXCL12/13), and cytokines (IL-6, IL-7, IL-33). Potential triggers include viral infection, genetic susceptibility, environmental factors, and hormonal influences. Amplification: B-cell hyperactivity is the central amplification axis, characterised by hypergammaglobulinaemia, elevated serum BAFF, and expanded circulating plasmablasts producing anti-SSA/Ro and anti-SSB/La autoantibodies; type I IFN further amplifies this circuit. Chronicity: fibroblasts acquire a pro-inflammatory “immunofibroblast” phenotype producing CXCL12, CXCL13, and IL-6, leading to extracellular matrix remodelling, fibrosis, acinar loss, and treatment resistance. Lymphomagenesis: ectopic lymphoid structures with germinal centres provide a permissive niche for MALT lymphoma with potential transformation to DLBCL; recognised clinical predictors of this lymphomagenic transition include cryoglobulinaemic vasculitis, persistent parotid swelling, and hypocomplementaemia (low C4). These events are subsequently reorganised in our review along three operational axes (Section 2).

We propose that SjD is most usefully organised around three dominant biological axes. Each captures a coherent constellation of pathogenic mechanism, biomarker signature, histopathological substrate, and therapeutic target. The axes overlap, are not mutually exclusive within an individual patient, and should be understood as dominant biological drivers rather than mutually independent disease subtypes. Their utility lies in providing a small enough number of categories to be clinically operational, while preserving the genuine biological heterogeneity that has defeated previous one-size-fits-all trial designs. Figure 1 provides a schematic overview of the pathogenic events these axes integrate, organised by their proposed roles as initiators, amplifiers, drivers of chronicity, and pathways to lymphomagenesis in sjD.

### 2.1. Axis 1: The Interferon-Driven Systemic Axis

A type I IFN transcriptional signature—increased expression of interferon-stimulated genes (ISGs) including MX1, IFI44L, ISG15, and IFIT1—is detectable in approximately 60% of patients and correlates with systemic disease activity and extraglandular manifestations [27,28]. Plasmacytoid dendritic cells (pDCs) produce type I IFN, which in turn upregulates BAFF, augments antigen presentation, promotes epithelial apoptosis, and amplifies autoantibody production [29]. The IFN signature is thus both a marker of and a driver of systemic inflammation, with self-reinforcing JAK–STAT signalling at its core. Salivary gland epithelial cells participate actively in this axis: they express functional Toll-like receptors (TLR3, TLR7, TLR9), produce IFN-α and IFN-β, and release autoantigens (Ro/SSA, La/SSB) following apoptosis, positioning the epithelium as both target and amplifier of IFN-driven inflammation [20,23,30,31,32,33,34,35,36,37].

The IFN axis is therapeutically tractable through pathway convergence on JAK–STAT, but SjD-specific phase III evidence is currently absent. Translation rests largely on extrapolation from systemic lupus erythematosus, where anifrolumab achieved phase III success in IFN-high patients [38,39], and on small SjD trials of JAK inhibition and low-dose IL-2 [40]. This reliance on cross-disease inference is, however, beginning to be addressed directly: deucravacitinib, a selective allosteric TYK2 inhibitor that blocks signalling of type I IFN alongside IL-12 and IL-23, is approved in psoriasis and has advanced to replicate phase III trials in SLE (POETYK SLE-1/2), and is now under evaluation in a dedicated phase III trial in active SjD (NCT05946941)—offering the prospect of axis-1-specific evidence in place of continued extrapolation [41]. The biomarker side of the axis is more mature than the therapeutic side: IFN gene scores and the cell-surface surrogate SIGLEC-1 are analytically robust, reproducible across cohorts, and ready for trial enrichment, though a regulatory-qualified harmonised assay is lacking. A central unresolved question is whether IFN-high states represent stable endotypes or transient inflammatory phases [42,43,44]; cross-sectional data suggest medium-term stability, but prospective longitudinal characterisation is sparse.

### 2.2. Axis 2: The B-Cell/Lymphoproliferative Axis

B-cell hyperactivity is the most thoroughly validated immunological feature of SjD and the only axis for which positive SjD phase III evidence currently exists. The defining biology includes hypergammaglobulinaemia in up to 80% of patients, production of anti-Ro/SSA and anti-La/SSB autoantibodies, expanded circulating plasmablasts, and elevated serum BAFF [45,46,47,48,49]. Within affected glands, B cells organise into ectopic lymphoid structures (ELS) with germinal centre-like architecture, enabling local affinity maturation and creating the permissive niche from which MALT and diffuse large B-cell lymphomas emerge [50,51,52,53,54,55]. The continuum from autoimmunity to lymphoproliferation is the most clinically consequential feature of this axis: lifetime lymphoma risk reaches 5–10%, and risk is concentrated in patients with persistent parotid swelling, cryoglobulinaemic vasculitis, hypocomplementaemia (particularly low C4), rheumatoid factor positivity, and biopsy features of focus score ≥ 4 with ectopic germinal centres [56,57,58,59,60,61,62,63,64]. These markers carry sufficient negative predictive value to drive structured surveillance and to identify patients in whom early, intensified B-cell-directed therapy is most likely to be biologically and clinically meaningful. It is important to emphasise that these features function as prognostic surveillance markers that stratify the long-term risk of lymphomagenesis; they are conceptually distinct from predictive biomarkers of therapeutic response. The two roles should not be conflated, and a marker validated for lymphoma-risk surveillance should not be assumed, without dedicated evidence, to predict response to a given therapy when used for trial enrichment.

Therapeutic translation on this axis is now substantive. Ianalumab, whose dual mechanism combines inhibition of BAFF–BAFF receptor (BAFF-R) signalling with antibody-dependent cellular cytotoxicity (ADCC)-mediated depletion of BAFF-R-expressing B cells, achieved its ESSDAI primary endpoint in the replicate phase III NEPTUNUS-1 and NEPTUNUS-2 trials [17,18,19]; this is, to our knowledge, the first replicated phase III evidence in SjD demonstrating that targeted B-cell modulation can translate into clinically meaningful disease activity reduction [65,66] (these efficacy data are, at the time of writing, available only as a late-breaking congress abstract and company-reported results, with the full peer-reviewed publication awaited). Iscalimab (anti-CD40) produced a significant dose–response on ESSDAI in TWINSS phase 2b [20], and dazodalibep (anti-CD40L) showed efficacy in a phenotype-defined subset (see Axis 3) [22]. Nipocalimab, an FcRn (neonatal Fc receptor) inhibitor acting on the autoantibody compartment, produced an early phase 2 signal in DAHLIAS [21]. Additional FcRn-targeted agents are advancing in parallel: efgartigimod met its primary endpoint in the phase 2 RHO proof-of-concept study and has progressed to the phase III UNITY trial, while the next-generation FcRn antagonist IMVT-1402 is in phase 2b evaluation in anti-Ro/SSA-positive SjD [67,68]. Rituximab continues to produce biological and histopathological responses even where primary endpoints have not been met [14,15,69,70], supporting its role as a component of combination strategies rather than as a standalone disease-modifying therapy. BTK inhibition (remibrutinib) and additional B-cell-directed agents are in earlier development [71,72,73].

The implication is direct: B-cell-axis biomarkers—CXCL13, plasmablasts, BAFF, biopsy features—should not be regarded as supplementary measurements but as the enrichment criteria for the next generation of trials in this population.

### 2.3. Axis 3: The Symptom/Fibro-Structural Axis

A substantial subset of patients present with high symptom burden—fatigue, widespread pain, sicca, and disability—in the absence of active systemic inflammation or organised lymphoid pathology. These patients are characterised by high ESSPRI, low ESSDAI, and frequent fibromyalgia overlap. Critically, this symptom-dominant phenotype should not be equated with advanced structural or fibrotic glandular change: even seronegative, symptom-predominant patients typically retain a positive focus score indicating ongoing immune-mediated glandular inflammation, do not consistently demonstrate greater glandular fibrosis or fatty infiltration on minor salivary gland biopsy, and frequently respond to secretagogues—indicating preserved and at least partially reversible exocrine function. We therefore interpret the dominant driver in this group as central sensitisation and neuroimmune dysregulation rather than fixed structural damage. We group this presentation with a related and partially overlapping phenotype of established structural damage in late disease, in which progressive stromal remodelling, immunofibroblast activation, and irreversible architectural loss explain treatment-resistant sicca despite immunomodulation [74,75,76,77]. We acknowledge that the symptom-dominant and fibro-structural phenotypes are mechanistically distinct—central sensitisation and neuroimmune dysregulation in the former, structural and stromal pathology in the latter—but we group them as a third axis because both share the defining feature of dissociation from active inflammation. With further evidence these may warrant separation, but in current data the dominant clinical implication is the same: agents directed at active inflammation will not work, and trials enrolling these patients into immunomodulation studies will fail at the population level even when the therapy is biologically correct for other patients.

The proof-of-principle for axis-aware design on this axis comes from dazodalibep, which improved ESSPRI in a prospectively defined phase 2 cohort selected for high symptom burden and low systemic activity [22]. This is currently the only positive trial in symptom-dominant disease and establishes that phenotype-matched enrolment can reveal therapeutic effect even where unstratified trials of the same mechanism would likely have failed. This signal should, however, be interpreted with mechanistic caution: dazodalibep is a CD40L antagonist, and its benefit in symptom-dominant patients plausibly reflects modulation of CD40–CD40L co-stimulatory B–T-cell interactions rather than an exclusively neuro-symptomatic mode of action, indicating that residual immune mechanisms may remain operative even in this low-systemic-activity group. Beyond this signal, evidence-based options remain limited. Non-pharmacological strategies (exercise, cognitive behavioural therapy, neuromodulators for neuropathic pain), muscarinic agonists for residual exocrine function, and individualised symptom management constitute the current standard. The endpoint architecture for this axis is the least developed: ESSDAI is insensitive by design, and ESSPRI alone is susceptible to placebo response and to confounding by non-disease determinants of symptom burden. Composite endpoints with patient-reported, objective glandular function, and structural components (the Sjögren’s Tool for Assessing Response [STAR] and the Composite of Relevant Endpoints for Sjögren’s Syndrome [CRESS]) are likely to be most informative here [78,79,80,81], but axis-specific validation is ongoing.

### 2.4. Why Three Axes, and What They Are Not

The three axes are not subtypes. Most patients show features of more than one, and the dominant axis can shift over time—for example, an IFN-high young patient may evolve toward B-cell-driven pathology with ELS formation, and any patient with longstanding disease accrues fibro-structural damage regardless of initial axis. The axes are also not a complete taxonomy of SjD biology; T-cell contributions (Th17, T follicular helper, regulatory T cells) [82,83,84,85] and innate immune circuits cross-cut all three. What the axes provide is an operational vocabulary: a small enough number of categories to be applied in the clinic, in trial enrolment, and in interpreting new evidence; and large enough to capture the genuine biological heterogeneity that has previously been diluted into single-arm population-wide trials. The remainder of this review is organised around testing this framework against the available data on stratification, biomarkers, therapeutics, and trial design.

## 3. Stratification: Mapping Clinical and Molecular Clusters onto the Three Axes

The literature on SjD stratification has produced several independent classifications: symptom-based clinical clusters [24,25,26], molecular clusters derived from transcriptomic and multi-omics data [42,86,87,88,89,90], and histopathological prognostic groupings [91,92,93]. A central question is whether these classifications identify the same patients from different vantage points or capture genuinely distinct dimensions of disease biology.

Symptom-based clustering by Tarn et al. identified four reproducible subgroups: low symptom burden, high symptom burden across all domains, dryness-dominant with fatigue, and pain-dominant with fatigue [94]. Post hoc reanalysis of rituximab trial data demonstrated differential treatment effects across these clusters [94], independently supported by McCoy et al. [41]. Nguyen et al. extended this research in the Paris-Saclay and ASSESS cohorts, describing three subgroups: a B-cell-active group with autoantibodies, elevated CXCL13, and increased lymphoma risk; a high systemic activity group; and a low systemic/high symptom group with features overlapping fibromyalgia-like syndromes [26]. Molecular stratification by Soret et al. identified four reproducible clusters using transcriptomic, epigenomic, and immunophenotypic integration: IFN-high, B-cell-active, myeloid-driven, and low-inflammatory [42,95,96] (Table 1).

These independent classifications map, imperfectly but recognisably, onto the three-axis framework. The IFN-high molecular cluster of Soret et al. and the high systemic activity group of Nguyen et al. correspond predominantly to Axis 1. The B-cell-active clusters of both Soret et al. and Nguyen et al., together with biopsy-defined high focus score and ectopic germinal centre patients, populate Axis 2. The low symptom/low inflammatory and high symptom/low inflammatory groups of Tarn and Nguyen, together with the fibrotic late-stage phenotype, populate Axis 3. The myeloid-driven cluster of Soret et al. does not map cleanly to a single axis and likely represents either a transitional state or a fourth biological pattern that requires further characterisation—a useful illustration that the three-axis framework is an operational simplification, not a final ontology.

Two limitations of current stratification work deserve emphasis. First, almost all stratification evidence is cross-sectional; longitudinal stability of cluster assignment is largely unknown. Whether a B-cell-active patient at year 1 remains B-cell-active at year 5, or transitions toward fibro-structural pathology, has direct implications for trial design but remains essentially unanswered. Second, multi-omics profiling—the most biologically informative stratification approach—remains a research tool. Translating molecular clusters into pragmatic, scalable assays usable outside specialised centres is a precondition for routine deployment, and current evidence supports IFN gene scores, CXCL13, and biopsy-based features as the realistic short-term enrichment tools while broader molecular infrastructure matures [86,97,98].

## 4. Translational Biomarkers: An Axis-Aware Decision Framework

**Figure 2:**
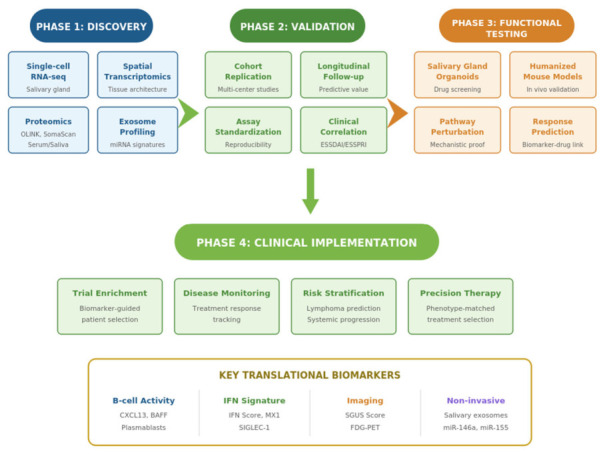
Translational biomarker pipeline. Discovery (multi-omics platforms) → analytical validation → clinical validation in cohort studies → regulatory qualification → clinical implementation. Different biomarkers serve different purposes under the BEST Framework: diagnostic classification, prognostic stratification, predictive enrichment for clinical trials, and pharmacodynamic monitoring of treatment response. Confusion over these intended uses has contributed substantially to the negative trial record in SjD patients.

The biomarker literature in SjD has been substantial but, until recently, has lacked structure. Markers have been deployed exploratorily and descriptively, often without explicit reference to their intended use under the FDA–NIH BEST (Biomarkers, EndpointS, and other Tools) Framework (diagnostic, prognostic, predictive, pharmacodynamic, monitoring). Two consequences follow: prognostic markers have been applied as predictive markers in trial enrichment, and pharmacodynamic markers have been treated as evidence of clinical benefit. Both errors have contributed to the negative trial record. We propose that biomarkers in SjD should be characterised by three properties simultaneously: which axis they serve, their BEST category, and their translational readiness (Table 2, Figure 2).

Axis 1 (IFN-driven) biomarkers are the most analytically mature in the field. IFN gene scores derived from ISG expression panels distinguish IFN-high from IFN-low patients robustly across cohorts [27,28,43,44], support enrichment for IFN-directed therapy, and act as pharmacodynamic readouts of pathway suppression. SIGLEC-1 (CD169) on circulating monocytes provides a pragmatic flow-cytometry-based surrogate measurable in routine clinical immunology laboratories [99,100]. The remaining gap is a regulatory-qualified, harmonised assay platform; this is achievable but not yet realied. As an enrichment biomarker, IFN scores are ready for prospective use in trials of JAK inhibitors, anti-IFNAR therapy, and Treg-directed strategies.

Axis 2 (B-cell/lymphoproliferative) biomarkers are the most clinically validated and the most directly tied to existing therapeutics. Serum CXCL13 correlates reproducibly with ESSDAI, biopsy severity, and lymphoma risk; it declines in response to B-cell-targeted therapy, supporting use as both enrichment and pharmacodynamic readout [101,102,103,104]. Plasmablast frequencies provide dynamic pharmacodynamic measurement of B-cell depletion. Biopsy-based features—focus score ≥ 4 and ectopic germinal centres—provide the strongest prognostic signal for lymphoma development and identify the patients in whom early intensified intervention is biologically justified [56,57,58,59,60,91,92,105,106,107]. Salivary gland ultrasound (SGUS; OMERACT-scored) offers a non-invasive structural complement with growing reproducibility, suitable for screening, prognostication, and treatment response monitoring [108,109,110,111]. These markers, collectively, are the most enrichment-ready in the field and should now be considered standard for trial enrolment on Axis 2. 

Axis 3 (symptom/fibro-structural) biomarkers are the least developed. ESSPRI is the established patient-reported instrument but is susceptible to placebo response and to non-disease confounders. SGUS structural scores, objective salivary and lacrimal flow measurements, and validated fatigue/pain instruments are required for adequate enrolment and endpoint capture on this axis. Whether central sensitisation biomarkers or fibrosis-specific imaging will mature into trial-ready tools remains open. The pragmatic recommendation for current trial design on Axis 3 is to combine high baseline ESSPRI with documented low ESSDAI, structural evidence on SGUS where available, and explicit exclusion of patients with active systemic features that would belong on Axes 1 or 2.

## 5. Therapeutic Implications: Axis-Matched Mechanism and Trial Design

**Figure 3:**
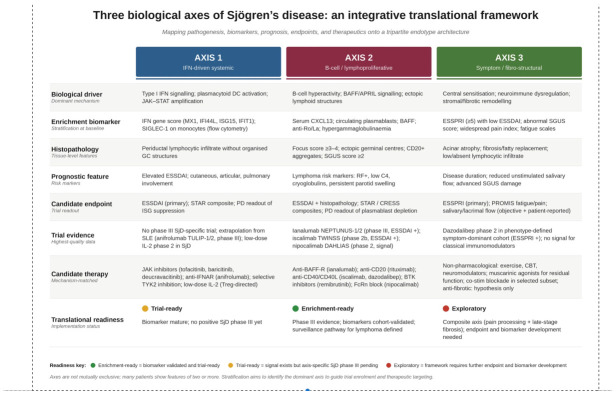
Three biological axes of Sjögren’s disease: an integrative translational framework. Each axis (IFN-driven systemic; B-cell/lymphoproliferative; symptom/fibro-structural) is mapped onto biological driver, enrichment biomarker, histopathology, prognostic feature, candidate endpoint, available trial evidence, candidate therapy, and translational readiness rating. The framework is operational rather than ontological: axes overlap within individual patients, and the dominant axis can shift over time. Readiness ratings (green = enrichment-ready, yellow = trial-ready signal pending phase III, red = exploratory) reflect current ability to deploy biomarker-enriched, mechanism-matched trial designs.

The trial record in SjD becomes coherent once viewed through the three-axis lens. The negative trials of rituximab (TRACTISS, TEARS) and abatacept (ASAP-III) enrolled biologically heterogeneous populations spanning all three axes and applied endpoints (fatigue VAS, oral dryness VAS, ESSDAI in moderate disease) poorly matched to the dominant axis in many enrolled patients [14,15,16,112,113,114,115,116,117]. Persistent BAFF elevation following CD20 depletion further limited durability on Axis 2 [47,48]. Where stratification has been attempted—explicitly in NEPTUNUS, dazodalibep, and TWINSS, implicitly in BELISS—signal has emerged (Table 3 and Figure 3).

### 5.1. Axis 1: Therapeutic Targeting of IFN Signalling

JAK–STAT signalling provides a mechanistically rational target for the IFN-driven axis, and convergence of IFN-α/β, IL-6, and other cytokines on this pathway supports broad anti-inflammatory effect. SjD-specific evidence remains early-phase: small studies of JAK inhibitors have shown biological signal without definitive clinical endpoint achievement, and the most relevant phase III data come from anifrolumab in SLE [38,39], where IFN-high enrichment was central to success. Selective TYK2 inhibition is in earlier development. Low-dose IL-2, with its Treg-restoring mechanism, achieved a positive small phase 2 signal in SjD [40] and may be particularly relevant for the IFN-high inflammatory subset. The translational priority on Axis 1 is straightforward: design a phase III SjD trial enriched by IFN signature, with ESSDAI as the primary endpoint and ISG suppression as the pharmacodynamic readout, testing JAK inhibition or anti-IFNAR therapy. The biomarker and endpoint infrastructure are ready; but the trial has not yet been performed. 

### 5.2. Axis 2: Therapeutic Targeting of B-Cell Hyperactivity

This is the axis on which the strongest contemporary evidence rests. Ianalumab’s success in NEPTUNUS-1 and NEPTUNUS-2 [17,18,19] establishes BAFF-R blockade with enhanced cellular cytotoxicity as currently the most advanced investigational targeted therapy in SjD—having demonstrated positive results in two replicate phase III trials (currently reported at congress and company level and not yet approved by regulatory authorities) and representing a promising candidate to become the first approved disease-modifying treatment for the disease; it also establishes that biology-aware enrolment (active systemic disease, ESSDAI ≥ 5) and ESSDAI as a primary endpoint can detect treatment effect when applied to an axis-appropriate population. Iscalimab (anti-CD40, TWINSS phase 2b) [20] and dazodalibep (anti-CD40L, phase 2) [22] establish co-stimulatory blockade as a viable axis-2 strategy pending phase III confirmation. Nipocalimab (FcRn inhibition, DAHLIAS phase 2) [21] provides early evidence for an autoantibody-clearance approach. BTK inhibition (remibrutinib) is at proof-of-concept [72]. Rituximab, despite negative primary endpoints in TRACTISS and TEARS, continues to produce biological and histopathological responses; its role is most plausibly as a component of combination strategies—for example, sequential or concurrent BAFF inhibition to address rebound—rather than as a standalone agent [14,15,69,70].

The clinical implication is direct: patients with active systemic disease, CXCL13 elevation, and biopsy-defined B-cell pathology should now be considered candidates for axis-2 directed therapy in trials and, as regulatory pathways evolve, in clinical care. Surveillance for lymphoproliferation in patients with focus score ≥ 4, ectopic germinal centres, RF positivity, low C4, or cryoglobulinaemia should be structured and routine [56,57,58,59,60,61,62,63,64,120,121]; whether intensified B-cell-directed therapy reduces lymphoma incidence is an important hypothesis warranting prospective evaluation.

### 5.3. Axis 3: Therapeutic Targeting in Symptom-Dominant and Fibro-Structural Disease

This axis is where the dominant lesson from the trial record is restraint rather than escalation. The dazodalibep phase 2 programme [22] is best interpreted as a two-population signal rather than as a purely symptom-dominant result: its systemic-activity cohort is relevant to Axis 2/mixed inflammatory disease, whereas the low-systemic, symptom-dominant cohort supports the possibility that selected mechanisms—CD40L blockade in this case—can reach benefit even where active systemic inflammation is limited, possibly by modulating neuroimmune circuits that are not captured in conventional inflammation biomarkers. Whether this extends to other co-stimulation or anti-cytokine approaches is unknown. For the broader axis-3 population—particularly patients with established fibrotic damage—the trial record is uniformly negative, and there is little reason to believe that immunomodulation will reverse irreversible architecture. Non-pharmacological symptom management, exercise, cognitive behavioural therapy, neuromodulators for neuropathic pain, and muscarinic agonists for residual exocrine function constitute the current evidence base [122]. Anti-fibrotic strategies remain hypothesis-generating. 

The implication for trial design is two-fold. First, axis-3 patients should generally be excluded from trials targeting active inflammation, both to avoid diluting signal in the active arms and to avoid generating spurious negative results that are attributed to therapeutic failure. Second, axis-3-specific trial designs are needed, with axis-3-appropriate enrolment (high ESSPRI, low ESSDAI, structural evidence), endpoint architecture (ESSPRI, fatigue/pain instruments, objective glandular function), and likely longer duration to detect symptomatic response.

## 6. A Precision-Medicine Roadmap for SjD

The infrastructure for axis-aware trial design is largely in place. Enrolment biomarkers (IFN scores and SIGLEC-1 for Axis 1; CXCL13, plasmablasts, biopsy features, and SGUS for Axis 2; ESSPRI plus structural and objective glandular function measures for Axis 3) are available, and most can be deployed in specialised centres with existing assays. Composite endpoints—STAR and CRESS—have been developed and partially validated to capture the multidimensional treatment effects that ESSDAI and ESSPRI individually miss [78,79,80,81,123,124,125,126,127]. The remaining is operational rather than conceptual and falls into four areas (Table 4).

**Endpoint validation and consensus.** STAR and CRESS require continued prospective validation, including direct comparison against ESSDAI and ESSPRI in trials of axis-matched therapies. Integration of composite outcomes should now be considered standard for phase II and III SjD trials targeting specific biological pathways [128,129,130]. Axis-specific endpoint emphasis is reasonable: ESSDAI-dominant for Axes 1 and 2, ESSPRI-dominant with structural complements for Axis 3.

**Biomarker qualification and access.** Regulatory qualification of IFN gene scores and CXCL13 for trial enrichment requires early and structured engagement with regulatory agencies, with the anifrolumab–IFN score pathway in SLE as the most directly relevant precedent [131,132]. Pragmatic deployment will need affordable, scalable assays usable outside research settings; this is the largest single obstacle to broad axis-aware care.

**Health economics.** Formal cost-effectiveness analyses comparing biomarker-guided versus empirical treatment selection are absent. The economic argument for stratification is compelling—avoidance of ineffective biologic therapies, targeted use of high-cost agents, potential reduction of lymphoma morbidity—but it requires modelling embedded in future trials.

**Prospective validation of axis assignment.** Most stratification evidence is cross-sectional. Whether axis assignment is stable over months to years, whether patients transition predictably between axes, and whether axis-guided therapy improves outcomes over empirical care are open questions requiring prospective, randomised strategy trials. In the interim, biomarker-enriched designs, adaptive platforms with axis-stratified arm selection, and large observational cohorts linking axis assignment to real-world outcomes are feasible and informative. Notably, several recent trials have already begun to embed axis-aware enrolment: the iscalimab TWINSS programme, for example, incorporated a separate high-symptom/low-systemic-activity cohort alongside its systemically active population, providing a concrete precedent for the stratified designs proposed here.

## 7. Conclusions

We propose that the repeated negative trials in SjD may, to a substantial degree, reflect treatment mistargeting rather than intrinsic treatment resistance. Two decades of negative trials reflect biologically naïve design—heterogeneous populations, endpoints divorced from mechanism, and therapies applied without reference to a dominant pathway in the patient enrolled—rather than failure of immunomodulation as a strategy. When trial design has been aligned to biology, as reported for NEPTUNUS, TWINSS, DAHLIAS, and the dazodalibep two-population phase 2 programme, therapeutic signal has emerged, although several of these datasets remain abstract-level or recently published and require careful citation. A three-axis architecture—IFN-driven systemic, B-cell/lymphoproliferative, and symptom/fibro-structural—provides a parsimonious, clinically operational framework that maps coherently onto pathogenesis, biomarkers, endpoints, prognostic features, and therapeutic targets. The infrastructure to deploy this framework now exists; what remains is to apply it consistently in prospective trial design, biomarker qualification, and clinical care. If pursued, SjD may transition from a recurring example of translational failure into a model for precision medicine in heterogeneous autoimmune disease—with lessons for other conditions, including lupus, systemic sclerosis, and inflammatory myopathies, where comparable biological heterogeneity has contributed to translational frustration—while recognising that each of these diseases also presents distinct trial design challenges of its own, such as the need to account for life-threatening organ involvement (for example, lupus nephritis), which shape their therapeutic priorities differently.

## Figures and Tables

**Table 1:** Molecular and clinical stratification studies in SjD: mapping onto the three-axis framework.

Study	Design	n	Method	Clusters Identified	Mapping to Axes
Soret et al., 2021 [23]	Cross-sectional	304	Multi-omics (transcriptomic, epigenomic, flow cytometry)	IFN-high; B-cell-active; myeloid-driven; low-inflammatory	Axis 1; Axis 2; uncertain; Axis 3
Tarn et al., 2019 [24]	Cross-sectional + post hoc RCT reanalysis	608	Symptom-based clustering	Low symptom; high symptom; dryness-dominant + fatigue; pain-dominant + fatigue	Axis 3 dominantly; mixed
McCoy et al., 2022 [25]	Cross-sectional	International multi-cohort	Symptom-based	Independently replicated Tarn clusters	Axis 3 dominantly
Nguyen et al., 2024 [26]	Cross-sectional (Paris-Saclay) + prospective (ASSESS)	240	Cytokine + serology cluster	B-cell-active; high systemic; low systemic/high symptom	Axis 2; Axis 1; Axis 3

Abbreviations: IFN, interferon; RCT, randomised controlled trial; SjD, Sjögren’s disease.

**Table 2:** Translational biomarkers in SjD: a decision matrix.

Biomarker	Axis Served	BEST Category	Readiness	Scalability	Enrichment Potential	Key Implementation Barrier
IFN gene score	1	Predictive, pharmacodynamic	Trial-ready	Specialised labs	High	Lack of harmonised regulatory-qualified assay
SIGLEC-1 (CD169) on monocytes	1	Predictive, pharmacodynamic	Trial-ready	Routine flow cytometry	High	Inter-laboratory protocol standardisation
Serum CXCL13	2	Predictive, pharmacodynamic, prognostic	Enrichment-ready	Standard ELISA	High	Cut-off harmonisation
Plasmablast frequency	2	Pharmacodynamic	Trial-ready	Flow cytometry	Moderate (PD use)	Panel standardisation
Serum BAFF	2	Predictive, pharmacodynamic	Trial-ready	Standard ELISA	Moderate	Threshold definition
Focus score ≥ 4	2	Prognostic	Established	Invasive biopsy	High (in lymphoma-prone cohorts)	Invasiveness; interobserver variability
Ectopic germinal centres	2	Prognostic	Established	Specialised IHC	Very high (lymphoma risk)	Limited routine availability
Salivary gland ultrasound (OMERACT)	2, 3	Diagnostic, prognostic, monitoring	Clinically implementable	Bedside	High	Operator training and certification
ESSPRI	3	Patient-reported endpoint	Established	Self-administered	Moderate	Placebo susceptibility; non-disease confounding
Multi-omics cluster assignment	1, 2, 3	Predictive (exploratory)	Research-grade	Specialised platforms	Potentially very high	Pragmatic assay development; cost; access

Abbreviations: BEST, Biomarkers, EndpointS, and other Tools framework (FDA–NIH); ELISA, enzyme-linked immunosorbent assay; IHC, immunohistochemistry; OMERACT, Outcome Measures in Rheumatology; PD, pharmacodynamic; SjD, Sjögren’s disease.

**Table 3:** Trials in Sjögren’s disease, mapped to the three axes.

Trial	Agent	Target	Phase	n	Primary Endpoint	Outcome	Dominant Axis	Evidence Level/Source
TRACTISS (Bowman et al., 2017) [118]	Rituximab	CD20	III	133	Fatigue + dryness VAS	Not met	2 (heterogeneous enrolment)	Phase III; peer-reviewed full publication (primary endpoint not met)
TEARS (Devauchelle-Pensec et al., 2014) [15]	Rituximab	CD20	III	120	Global VAS	Not met	2 (heterogeneous enrolment)	Phase III; peer-reviewed full publication (primary endpoint not met)
ASAP-III (van Nimwegen et al., 2020) [16]	Abatacept	CD80/86	III	80	ESSDAI	Not met	Mixed; ESSDAI insensitive	Phase III; peer-reviewed full publication (primary endpoint not met)
BELISS (De Vita et al., 2015) [119]	Belimumab	BAFF	II	30	ESSDAI	Met	2	Phase II; peer-reviewed full publication (open-label)
NEPTUNUS-1 & -2 (Mariette/Dörner et al., 2025) [17,18,19]	Ianalumab	BAFF-R + ADCC	III	275 + 504	ESSDAI	Met	2	Phase III; congress abstract/company-reported—peer-reviewed full publication pending
TWINSS (Fisher et al., 2023) [20]	Iscalimab	CD40	IIb	173	ESSDAI (dose–response)	Met	2	Phase 2b; congress abstract
DAHLIAS (Noaiseh, G.; et al., 2025) [21]	Nipocalimab	FcRn	II	163	ClinESSDAI at week 24	Met at 15 mg/kg; not significant at 5 mg/kg	2	Phase II; peer-reviewed full publication
Dazodalibep phase 2 (St Clair et al., 2024) [22]	Dazodalibep	CD40L	II	Two cohorts	Systemic endpoint in cohort 1; ESSPRI in cohort 2	Met in both cohorts; cohort 2 supports symptom-dominant enrichment	2/3 (cohort-dependent)	Phase II; peer-reviewed full publication (prospectively defined cohorts)
Remibrutinib PoC (Dörner et al., 2022) [72]	Remibrutinib	BTK	II	—	ESSDAI	Signal	2	Phase II; congress abstract (proof-of-concept)
Low-dose IL-2 (He et al., 2022) [69]	IL-2	Treg expansion	II	—	ESSDAI	Signal	1	Phase II; peer-reviewed full publication (small RCT)

Abbreviations: ADCC, antibody-dependent cellular cytotoxicity; BAFF, B-cell activating factor; BTK, Bruton’s tyrosine kinase; ESSDAI, EULAR Sjögren’s Syndrome Disease Activity Index; ESSPRI, EULAR Sjögren’s Syndrome Patient Reported Index; FcRn, neonatal Fc receptor; PoC, proof-of-concept; VAS, visual analogue scale.

**Table 4:** An axis-based stratification framework for SjD clinical trials.

Axis	Enrolment Criteria	Enrichment Biomarker	Candidate Therapies	Primary Endpoint	Readiness
1—IFN-driven systemic	ESSDAI ≥ 5; documented extraglandular activity; IFN-high signature	IFN gene score; SIGLEC-1	JAK inhibitors; anti-IFNAR; selective TYK2; low-dose IL-2	ESSDAI; ISG suppression (PD)	Trial-ready
2—B-cell/lymphoproliferative	ESSDAI ≥ 5; anti-Ro/La positive; CXCL13 elevated; biopsy features (focus score ≥ 3)	CXCL13; plasmablasts; BAFF; focus score; ectopic GCs	Anti-BAFF-R (ianalumab); anti-CD40/CD40L; anti-CD20 (combination); BTK; FcRn	ESSDAI ± histopathology; STAR/CRESS composite	Enrichment-ready
3a—Symptom-dominant	ESSPRI ≥ 5; ESSDAI < 5; absence of organised lymphoid pathology; fibromyalgia overlap permitted	ESSPRI; widespread pain index; fatigue scales	Co-stimulation blockade (selected subset); non-pharmacological strategies; symptomatic management	ESSPRI; PROMIS instruments	Trial-ready (axis-3a specific designs)
3b—Fibro-structural	Long disease duration; advanced SGUS damage; reduced unstimulated salivary flow; biopsy fibrosis	SGUS structural score; objective glandular function	Anti-fibrotic strategies (hypothesis-generating); symptomatic management	Glandular function; structural endpoints	Exploratory
Lymphoma surveillance overlay (all axes)	RF+, low C4, cryoglobulin, persistent parotid swelling, focus score ≥ 4, ectopic GCs	Composite risk score (clinical + histological)	Structured surveillance; early B-cell-directed therapy in highest-risk subsets	Lymphoma-free survival	Implementable now

Abbreviations: GC, germinal centre; IFN, interferon; ISG, interferon-stimulated gene; PD, pharmacodynamic; PROMIS, Patient-Reported Outcomes Measurement Information System; RF, rheumatoid factor; SGUS, salivary gland ultrasound; SjD, Sjögren’s disease.

## Data Availability

No new data were created or analysed. Data sharing is not applicable.

## Abbreviations

SjD, Sjögren’s disease; ELS, ectopic lymphoid structures; IFN, interferon; ISG, interferon-stimulated gene; BAFF, B-cell activating factor; APRIL, a proliferation-inducing ligand; JAK, Janus kinase; STAT, signal transducer and activator of transcription; TYK2, tyrosine kinase 2; IFNAR, interferon-α/β receptor; ESSDAI, EULAR Sjögren’s Syndrome Disease Activity Index; ESSPRI, EULAR Sjögren’s Syndrome Patient Reported Index; STAR, Sjögren’s Tool for Assessing Response; CRESS, Composite of Relevant Endpoints for Sjögren’s Syndrome; GC, germinal centre; SGEC, salivary gland epithelial cell; SGUS, salivary gland ultrasound; BTK, Bruton’s tyrosine kinase; MALT, mucosa-associated lymphoid tissue; DLBCL, diffuse large B-cell lymphoma; Tfh, T follicular helper; Treg, regulatory T cell; RF, rheumatoid factor; ADCC, antibody-dependent cellular cytotoxicity; OMERACT, Outcome Measures in Rheumatology; ACR, American College of Rheumatology; EULAR, European League Against Rheumatism; VAS, visual analogue scale; CBT, cognitive behavioural therapy; PD, pharmacodynamic; PROMIS, Patient-Reported Outcomes Measurement Information System; BEST, Biomarkers, EndpointS, and other Tools framework; FcRn, neonatal Fc receptor; NPV, negative predictive value.
